# Local Recurrence After Lung Segmentectomy—Risk Factors and Future Directions: A Contemporary Review

**DOI:** 10.3390/cancers18162649

**Published:** 2026-08-17

**Authors:** András Buzás, Kitti Egyed, József Furák

**Affiliations:** 1 Department of Thoracic Surgery, National Korányi Institute of Pulmonology, HU-1122 Budapest, Hungary; 2 Department of Anaesthesiology and Intensive Therapy, Pest County Flór Ferenc Hospital, HU-2143 Kistarcsa, Hungary; 3 Department of Surgery, Thoracic Surgery, University of Szeged, HU-6725 Szeged, Hungary

**Keywords:** segmentectomy, local recurrence, early-stage non-small cell lung cancer

## Abstract

**Background:** Anatomical segmentectomy has increasingly emerged as an accepted surgical strategy for selected patients with early-stage non-small cell lung cancer (NSCLC), particularly following the JCOG0802/WJOG4607L and CALGB/Alliance 140503 randomized trials. Although these studies demonstrated oncologic equivalence—or even superiority in overall survival—compared with lobectomy in carefully selected tumors, concerns persist regarding increased locoregional recurrence after segmentectomy. Recent evidence suggests that recurrence is influenced not only by surgical technique but also by tumor biology, radiological characteristics, nodal assessment, and patient-specific factors. **Methods:** This narrative review critically summarizes contemporary evidence regarding local recurrence after anatomical segmentectomy for early-stage NSCLC. A literature review was performed using PubMed/MEDLINE database and recent thoracic surgical literature published primarily from 2015 onwards. Randomized trials, prospective and retrospective studies, meta-analyses, and expert consensus statements were included, with particular focus on recurrence patterns, tumor biology, radiological features, surgical factors, lymph node assessment, and emerging technologies. **Results**: Segmentectomy provides comparable overall survival to lobectomy in selected patients with small peripheral NSCLC; however, locoregional recurrence remains more frequent after segmentectomy. Major predictors of recurrence include pure-solid radiological appearance, high consolidation-to-tumor ratios, larger tumor size, STAS positivity, lymphovascular invasion, aggressive adenocarcinoma subtypes, inadequate lymph node assessment, and insufficient surgical margins. Hypermetabolic tumors on PET imaging and occult nodal disease further increase recurrence risk. Several studies identified delayed recurrence patterns occurring beyond 5 years after surgery. **Conclusions:** Local recurrence remains the principal oncologic limitation of segmentectomy. Contemporary evidence supports a shift from size-based toward biology-driven surgical decision-making integrating tumor morphology, metabolic activity, histopathological aggressiveness, STAS status, and nodal involvement into surgical planning.

## Simple Summary

Anatomical lung segmentectomy has become an increasingly accepted surgical treatment for selected patients with early-stage non-small cell lung cancer, particularly after recent randomized trials demonstrated survival outcomes comparable to lobectomy. However, concerns remain regarding the increased risk of local recurrence after segmentectomy. This review summarizes current evidence regarding the mechanisms, risk factors, and predictors of recurrence following segmentectomy. Contemporary studies suggest that recurrence is influenced not only by surgical technique, but also by tumor biology, including radiological appearance, histopathological subtype, spread through air spaces, nodal involvement, and metabolic activity on PET imaging. Technical factors such as adequate surgical margins, lymph node assessment, and anatomical complexity of segmentectomy also contribute to oncologic outcomes. Emerging technologies including navigational bronchoscopy, indocyanine green imaging, and three-dimensional surgical planning may further improve patient selection and surgical precision. A biology-driven approach may optimize the selection between segmentectomy and lobectomy in early-stage lung cancer.

## 1. Introduction

For decades, lobectomy with systematic lymph node dissection was the undisputed standard of care in thoracic surgery for early-stage non-small-cell lung cancer (NSCLC), largely based on the 1995 Lung Cancer Study Group trial, which showed a threefold increase in local recurrence and a significant survival disadvantage for sublobar resections in patients with T1-2 N0 NSCLC [1]. However, over the past decade, with technological advancements in imaging, lung cancer screening, early diagnosis, and minimally invasive and robotic surgical techniques, the role of segmentectomy in the management of early-stage NSCLC has evolved substantially and become the forefront topic in the field of thoracic surgery.

Two recent, pivotal randomized trials, JCOG0802/WJOG4607L and CALGB/Alliance 140503, have demonstrated oncologic equivalence—or even superiority in overall survival—between segmentectomy and lobectomy in selected patients with small (≤2 cm), clinically node-negative peripheral non-small-cell lung tumors [2,3]. Owing to the findings of these large randomized phase III trials, segmentectomy has progressively evolved from a compromised operation reserved for functionally limited patients into an intentional oncologic strategy for selected early-stage lung tumors. However, concerns remain regarding the higher incidence of locoregional recurrence following segmentectomy and the risk factors leading to recurrence in these patients. This narrative review aims to synthesize contemporary evidence on local recurrence after lung segmentectomy, focusing on data from randomized trials, post hoc analyses, prospective and retrospective observational studies. We examined the scientific literature for recurrence rates, patterns, timing, and risk factors, including tumor biology, surgical margins, and lymph node assessment, and compared results of segmentectomy and lobectomy patients to find a potential scientific margin to help day-to-day clinical decision-making and provide a better overall picture of this heavily debated topic in the field of thoracic surgery.

## 2. Materials and Methods

This narrative review was undertaken to summarize, critically evaluate, and synthesize the contemporary evidence on local recurrence after anatomical segmentectomy for early-stage NSCLC, together with the different recurrence patterns and risk factors, and compare them with those associated with lobectomy. A collective literature analysis was performed using the PubMed/MEDLINE database to identify relevant studies published between the beginning of 2015 and 2026 on the topic of local recurrence after anatomical segmentectomy for early-stage NSCLC. The search strategy included combinations of the following keywords: *recurrence after lung segmentectomy*, *segmentectomy vs. lobectomy*, *recurrence-free survival after segmentectomy*, *non-small-cell lung cancer*, *NSCLC*, *local recurrence*, *locoregional recurrence*, *segmentectomy and recurrence*, *STAS and recurrence*. Priority was given to randomized controlled trials, prospective studies, retrospective cohorts, propensity-matched analyses, expert consensus statements, and previous reviews in the recent literature evaluating recurrence patterns, risk factors, and long-term oncologic outcomes after anatomical segmentectomy for NSCLC. Studies were selected based on their relevance to recurrence patterns, local recurrence predictors, tumor biology, radiological characteristics, surgical technique, lymph node assessment, technological advances, and long-term oncologic outcomes following anatomical segmentectomy.

## 3. Evidence from Previous Randomized Controlled Trials

In the Japanese JCOG0802/WJOG4607L trial, published in 2022, 1106 patients with peripheral clinical stage IA ≤ 2 cm NSCLC and a consolidation-to-tumor ratio (CTR) > 0.5 were randomized to a lobectomy or segmentectomy group. The median follow-up period was 7.3 years. Segmentectomy was found to be superior to lobectomy in terms of overall survival (OS), with a 5-year OS of 94.3% after segmentectomy vs. 91.1% after lobectomy. Despite this survival advantage, the study found a significantly higher incidence of local relapse in the segmentectomy group, 10.5–11.2%, compared to 5.4–5.8% in the lobectomy group. The survival benefit was attributed to a reduction in deaths from non-cancer causes; for instance, due to better preservation of pulmonary function [2].

However, subsequent supplementary analyses of risk factors for locoregional relapse after segmentectomy in the JCOG0802/WJOG4607L study identified critical determinants of recurrence: pure-solid appearance on thin-section computer tomography (CT) and a surgical margin distance smaller than tumor size were found to be significantly associated with local recurrence and tumor location; complex anatomical resections were also identified as important factors [4].

The international CALGB/Alliance 140503 trial randomized 697 patients with peripheral cT1aN0 NSCLC ≤ 2 cm to a lobectomy or sublobar resection group (both segmentectomy and wedge resections) following intraoperative nodal confirmation and compared the two groups. This study found that sublobar resection was non-inferior to lobectomy for 5-year disease-free survival (DFS), 63.6% vs. 64.1%, respectively, with OS of 80.3% and 78.9%, respectively. Similarly to the JCOG0802 study, there was a trend toward higher recurrence in the sublobar group compared to the lobectomy group, 13.4% vs. 10.0%, respectively, although this finding did not reach statistical significance. However, in the sublobar group of 340 patients, 59.1% underwent wedge resection and only 37.9% anatomical segmentectomy, so the interpretation of these results remains limited for local recurrence following anatomical segmentectomy [3].

## 4. Incidence and Patterns of Local Recurrence from Retrospective Studies

In addition to the two landmark randomized phase III trials, numerous retrospective cohort studies have investigated the incidence, timing, and patterns of local recurrence following anatomical segmentectomy.

In their retrospective analysis, Wada et al. evaluated the locoregional relapse patterns of 115 patients with clinical stage IA NSCLC (≤2 cm for solid-dominant tumors (CTR > 0.5) and ≤3 cm for ground-glass opacity (GGO)-dominant tumors (CTR > 0.5)) after anatomical segmentectomy and compared the results with those of 292 patients who had undergone lobectomy with similar stage IA tumors (using the seventh edition of the TNM classification). They found that both OS and recurrence-free survival (RFS) were non-inferior after segmentectomy compared to those following lobectomy: the 5- and 10-year OS rates were 91.8% and 79.4% after segmentectomy, respectively, and 89.9% and 68.2% after lobectomy, while 5- and 10-year RFS rates after segmentectomy were 79.8% and 68.7%, respectively, and 77.2% and 61.2% after lobectomy. However, when examining locoregional relapses—local relapse defined as recurrence at surgical margins, in the bronchus, or in a preserved lobe, and regional relapse as occurrence in an ipsilateral lobe, a hilar or mediastinal lymph node, or the ipsilateral pleural space—they were found to be two times more frequent after segmentectomy than after lobectomy: 13.9% vs. 7.9%. Recurrence sites were the surgical margin, remnant lobe, ipsilateral lung, mediastinal lymph node, or ipsilateral dissemination, but no relapse occurred in the ipsilateral hilar lymph node. Interestingly, distant relapse patterns were more common after lobectomy, leading to a similar relapse rate (locoregional and distant combined) in both groups. However, the median time to locoregional relapse after segmentectomy was 1245.5 days, which was significantly longer than the 512 days after lobectomy, and locoregional relapse in the segmentectomy group was more frequent with solid-dominant tumors. Additionally, in the segmentectomy group, the CTR was significantly higher (solid-dominant tumors) in patients with local recurrence than in those without recurrence, while no significant differences were found in the tumor or solid component diameter per se. Interestingly, late recurrences frequently occurred beyond 4 years, even for solid-dominant tumors with diameters less than 1 cm [5].

Retrospective studies by Nomori et al. and Brascia and colleagues reported similarly higher local recurrence rates after segmentectomy [6,7]. Nomori and colleagues performed a retrospective analysis of a previous prospective cohort and found 8% recurrence (14 out of 179 enrolled patients) after segmentectomy for cT1N0M0 peripheral NSCLC (using the eighth edition of the TNM classification): 8 out of 14 cases were local relapses, but 6 of the 8 local relapse cases developed more than 5 years after segmentectomy, similarly to Wada et al.’s results, highlighting the necessity of long-term (beyond 5 years) follow-up after segmentectomies [6]. Brascia et al. recently published an article reporting a dual-center retrospective analysis of 209 patients with clinical stage IA1-IA2 NSCLC treated with segmentectomy and found local recurrence in 5.7% and regional recurrence in 8.6%. Among risk factors, lower diffusing capacity of carbon monoxide (DLCO) and higher tumor grade were significantly associated with local relapse (defined as tumor relapse at surgical margin, in preserved lobe, or in hilar lymph nodes), while pathological tumor size, lymphovascular invasion and visceral pleural invasion were significant predictors of regional recurrence (defined as recurrence in mediastinal or supraclavicular lymph nodes, in a hilar lymph node outside the operated lobe, or in a different ipsilateral lobe) [7]. Despite the relatively low number of enrolled patients and the limitations of the retrospective study design, both studies reported high 5- and 10-year OS and RFS, while for T1c tumors, the 10-year recurrence-free probability was only 69% (over 90% for in situ, microinvasive tumors and T1a-b tumors) [6,7]. Hafsa et al. carried out a low-volume retrospective single-center study of patients who had undergone segmentectomy (n = 27) or lobectomy (n = 68) for pathological stage IA3 tumors and demonstrated significantly worse 5-year DFS after segmentectomy compared to lobectomy (37% vs. 72%) [8]. On the other hand, Lopez et al. conducted a prospective multicenter cohort study of 3533 patients included in the registry of the Spanish VATS Group of the Spanish Society of Thoracic Surgery. Cases included 1004 lobectomies and 83 segmentectomies performed on patients with clinical stage IA tumors with a complete follow-up, excluding cases involving pure GGO tumors, neoadjuvant treatment, previous lung malignancy, synchronous tumors, or incomplete resection. They found similar 5-year OS for the segmentectomy and lobectomy groups, 73.1% and 73.5%, respectively, with a predominance of locoregional recurrence in the segmentectomy group (16.2% vs. 11.2%) and distant recurrence after lobectomy (12.5% vs. 7.5%). The mean tumor sizes of solid or mixed nodules among enrolled patients were 16.0 and 16.4 mm in the segmentectomy and lobectomy groups, respectively, while nearly 80% of the tumors were solid in the segmentectomy group and around 75% were solid in the lobectomy group [9]. Overall, these results should be interpreted with caution, as most studies included highly selected patient populations and differed considerably in patient selection criteria, lymph node assessment, surgical technique, pathological evaluation, and follow-up duration.

## 5. Potential Mechanisms of and Risk Factors for Local Recurrence

As presented below, multiple factors, including surgical, patient-specific, and technical characteristics, play a role in local recurrence after segmentectomy. In this section, we systematically examine each factor.

### 5.1. Tumor Size

The size of the tumor itself is the first and universally accepted limiting factor for segmentectomy eligibility; it is also a potential risk factor for recurrence. The previously mentioned retrospective studies found a link between tumor size and recurrence after segmentectomy. While a tumor size ≤ 2 cm is broadly considered the margin for safe segmentectomy, many researchers have found a smaller preferable diameter when analyzing recurrence rates. Vakouftsi and colleagues published a retrospective cohort study with 180 patients who had undergone segmentectomy for clinical stage IA1 and IA2 NSCLC and found recurrence in 18 patients (10%). In their logistic regression analysis, recurrence was fivefold higher in patients with tumor sizes ≥ 1.5 cm than in those with tumor sizes < 1.5 cm, and it was nearly threefold higher in patients with pathological upstaging (either T or N descriptor) than in those without [10]. Similarly, in their retrospective analysis of 353 patients who had undergone pulmonary lobectomy or segmentectomy with nodal dissection for NSCLC ≤ 2 cm (mean diameter of 16 mm) with a CTR between 0.5 and 1, Hattori et al. found that pure-solid appearance and maximum tumor size—14.3 mm of mean tumor size in their segmentectomy group—were significant predictors of recurrence [11]. Kamigaichi et al. carried out a prospective study with the data of 166 patients who underwent segmentectomy for clinical stage 0 or IA NSCLC (≤3 cm for GGO-dominant tumors or ≤2 cm for solid-dominant tumors) and identified only a CTR of 0.86 or higher as an independent predictor of recurrence [12]. These findings suggest that, even within the current ≤2 cm indication range, tumor size continues to influence oncologic outcomes and should be interpreted together with biological aggressiveness and radiological morphology.

### 5.2. Tumor Biology and Other Pathohistological Properties

Tumor biology and the presence of aggressive adenocarcinoma subtypes play an essential role in biology-driven surgical decision-making. Several studies have demonstrated that pathological aggressiveness of the tumor strongly influences local recurrence after segmentectomy. Xu and colleagues conducted a retrospective analysis of clinical and pathological data of 475 patients with stage IA lung adenocarcinomas and, in their univariate analysis, found that vascular invasion, STAS positivity, solid or micropapillary subtype, histological differentiation, and higher pathological TNM and CTR were statistically significantly linked with recurrence. In their multivariate analysis, pTNM classification, lymph node sampling (compared to no lymph node dissection), vascular invasion, and solid or micropapillary histological subtypes remained significantly correlated with recurrence [13]. Similarly, Brascia et al. identified a significant correlation between recurrence risk and larger tumor size, lingular resection, and lymphovascular invasion. While low DLCO and poor tumor differentiation predicted local recurrence in their analysis, tumor size and the presence of lymphovascular or pleural invasion predicted regional recurrence [7]. Additionally, Wu et al. conducted a retrospective analysis of 1504 patients with pathological stage I lung adenocarcinomas, and their results indicated that age over 62 years, visceral pleural invasion, micropapillary or solid predominant subtypes, and lymphovascular invasion were significant prognostic values for RFS. The administration of adjuvant chemotherapy in these high-risk-classified patients significantly improved RFS and OS, according to their findings [14]. While STAS positivity and vascular, lymphovascular, and pleural invasion have been linked with recurrence after segmentectomy, the role of perineural invasion—while associated with aggressive tumor biology and worse oncologic outcomes—after segmentectomy remains insufficiently characterized.

### 5.3. Nodal Involvement

Nodal involvement, adequate lymph node assessment, and room for possible understaging due to insufficient lymphadenectomy are also major concerns in segmentectomies. Tudor and colleagues reviewed 12 studies and more than 170,000 patients in their study and found that segmentectomy was associated with a significantly worse overall nodal upstaging rate compared to lobectomy (6.6% vs. 14.5%), with primarily lower N1 detection (3.7% vs. 13.3%). However, despite lower N1 detection, survival outcomes were comparable among patients who received adjuvant therapy for N1 or N2 disease after segmentectomy [15]. This survival outcome among N1-positive patients in the last study resonates with the European Society of Thoracic Surgeons (ESTS) consensus by Brunelli et al., where more than 75% of experts agreed that in cases of unexpected N1 or N2 positivity in the final pathological report, the patient should be referred for adjuvant chemotherapy and not for completion lobectomy [16]. Multiple studies support this consensus. Abdallat et al.’s analysis of 185 occult N1 cases of NSCLC showed that segmentectomy is associated with similar OS and RFS compared to lobectomy despite nodal involvement [17], while Nobel and colleagues did not find significantly different 5-year cumulative incidence of recurrence (CIR), OS, or rate of isolated locoregional recurrence (12% after segmentectomy vs. 13% after lobectomy with N1 or N2 disease) among 759 included patients who had undergone segmentectomy and were found to be node-positive on the final pathological report [18]. Adequate lymphadenectomy and thus the potential detection of nodal involvement, however, is fundamental. In another retrospective study of a high-volume center, Huang Q et al. demonstrated that after segmentectomy, patients with six or more harvested lymph nodes had a higher chance of nodal metastasis detection (9.4% vs. 1.5%), and a significantly higher 3-year RFS (90.2% vs. 73.7%) than those with fewer than six surgically removed lymph nodes. Their multivariate analysis identified six or more harvested lymph nodes as an independent predictor of improved RFS [19]. Similarly, in a low-volume retrospective study, Brown and colleagues identified a close parenchymal margin (1 cm or less) and the number of resected lymph nodes (median was 6) as significant modifiable predictors of recurrence [20]. In another study, Huang L et al. found a correlation between the preoperative maximum standardized uptake value (SUVmax) and nodal involvement. According to the findings of their retrospective study, SUVmax ≥ 4.5 (versus <4.5) was the only independent factor for nodal involvement among 478 patients with cIA1-2 NSCLC after segmentectomy (with an odds ratio of 3.5), highlighting a possible role for positron emission tomography (PET) scanning in predicting nodal involvement before surgery [21]. In another retrospective analysis of 357 clinical stage IA solid-dominant lung cancer patients, Handa et al. compared systematic/lobe-specific mediastinal lymphadenectomy to hilar lymphadenectomy and found that RFS was not significantly different between the two groups, but patients with mediastinal lymphadenectomy had significantly better OS compared to those with hilar lymphadenectomy: 90.8% vs. 82.1%, respectively. The median number of dissected nodes was 5 in the hilar group and 8 in the mediastinal group, and metastasis was found in 3.1% and 6.2%, respectively. In their propensity-score-matched analysis, the 5-year RFS was better in the mediastinal lymphadenectomy group than in the hilar group, 93.5% vs. 88.5%, respectively; OS was similarly higher in the mediastinal group, with 90.9% and 83.3%, respectively, and the mediastinal group experienced half the lymph node recurrence of the hilar group (2.3% vs. 4.7%). In summary, mediastinal lymphadenectomy provides better oncologic outcomes than hilar-only lymphadenectomy, and more patients are prescribed adjuvant therapy owing to better detection of lymph node involvement [22]. Moreover, supporting this conclusion, Mimae and colleagues recently published a study of 941 patients who underwent either segmentectomy (n = 332) or lobectomy (n = 609) for ≤2 cm solid-dominant peripheral NSCLC without clinical lymph node involvement. Locoregional recurrence of hilar lymph node metastasis was only 0.3% after lobectomy and 0.0% after segmentectomy, while locoregional recurrence of mediastinal lymph node metastasis was 3% and 1%, respectively. In this study, hilar and mediastinal lymph node dissection was performed, with means of 5 and 14 lymph nodes dissected in the segmentectomy and lobectomy groups, respectively [23]. These results further emphasize the role of adequate mediastinal–hilar lymphadenectomy even in cases of segmentectomy for solid-dominant tumors, while in cases of GGO-dominant lesions, because large-scale prospective data is not available, adherence to the International Association for the Study of Lung Cancer (IASLC) recommendations for intraoperative lymph node assessment for completeness of staging is still recommended.

### 5.4. Spread Through Air Spaces (STAS) Status

STAS is of utmost importance in recurrence after segmentectomies. It was incorporated into the 2015 World Health Organization lung tumor classification and the 8th edition of the IASLC staging guidelines due to its expanding prognostic value, and multiple studies have not only identified the role of STAS in local recurrence after segmentectomies but also revealed the brain to be a distant metastatic site even for small STAS-positive tumors [24,25]. Kadota and colleagues published a paper thoroughly reviewing tumor slides from 411 patients with small (≤2 cm) stage I lung adenocarcinomas. They found that the risk of 5-year cumulative incidence of recurrence (CIR) was significantly higher in patients with STAS-positive tumors than in those with STAS-negative tumors—42.6% vs. 10.9%, respectively. STAS was linked to a significantly higher risk of locoregional recurrence compared to STAS-negative tumors (5-year CIR, 22.2% vs. 4.1%). However, in their lobectomy group, the presence of STAS was not associated with an increased risk of any recurrence compared with the absence of STAS (5-year CIR, 12.7% vs. 9.5%) [26]. Similarly to Kadota’s results, the findings of Eguchi et al. also suggest that lobectomy mitigates STAS-associated recurrence risk: after analyzing the data of 1497 patients who had undergone lobectomy (n = 970) or sublobar resection (n = 527) for T1N0M0 lung adenocarcinomas, the authors found that sublobar resection was significantly associated with recurrence in patients with STAS-positive compared to STAS-negative tumors, and STAS-positive patients had a higher risk of locoregional recurrence, regardless of the margin-to-tumor ratio [27]. According to these results, STAS remains a highly aggressive pathohistological property, and if pre- or intraoperative verification is available, lobectomy provides better long-term outcomes. Still, it is important to mention that most available studies are retrospective and subject to selection bias. Furthermore, pathological assessment of STAS is not standardized across institutions, limiting direct comparison between studies.

### 5.5. Tumor Localization and Simple and Complex Segmentectomy

The preoperative prognostic value of tumor localization must also play a role in surgical decision-making when opting for segmentectomy or lobectomy. When analyzing data from the JCOG study to identify risk factors, Nakagawa and colleagues found that patients with tumors localized in the lingular, left S6, and left basal segments had local recurrence more often after segmentectomy than after lobectomy [4]. However, Teng et al. performed a retrospective analysis of 362 patients who had undergone either simple or complex segmentectomy and found that the latter was associated with better lymph node harvesting, but with no significant difference in either 3- or 5-year DFS or OS rates. Local recurrence did not significantly differ between the two groups, with 2.6% in the simple and 1% in the complex segmentectomy group [28]. Similarly, Handa et al. found that 5-year RFS rates were not significantly different in a retrospective analysis of 717 patients with hypermetabolic (SUVmax ≥ 2.5) clinical stage IA NSCLC who had undergone either complex segmentectomy or lobectomy [29]. While lingular, basal, and complex segmentectomies tend to have worse recurrence rates in the literature, further analysis of homogeneous patient groups is required to establish strong recommendations for selecting between simple and difficult single-segment resections, complex segment resections, and lobectomy.

### 5.6. Radiological Morphology (GGO–Semisolid–Solid) and CTR

The radiomorphology of the suspected nodule can aid in supporting or debating the role of segmentectomy in each case. Pure GGO nodules are more frequently diagnosed with low-dosage screening programs or are even diagnosed incidentally, and they are associated with in situ, minimally invasive or lepidic predominant patterns with better prognosis [30]. In contrast, pure-solid nodules on thin-section CT scans and maximum tumor size—according to the previously mentioned study by Hattori and colleagues—are significant predictors of locoregional recurrence. In their study, 87.5% of patients with locoregional recurrence had tumors with a pure-solid appearance on CT scans, and in their analysis of 212 patients with clinical T1 (≤2 cm) pure-solid nodules, the 3-year local RFS of the segmentectomy group was significantly worse than that of the lobectomy group (82.2% vs. 90.6%). The frequency of locoregional recurrence was significantly higher after segmentectomy compared to that in the lobectomy arm (20.7% vs. 8.2%) [11]. Logically, the most debated and analyzed group is the part-solid pulmonary nodules. Ye et al. carried out a retrospective analysis of 911 selected patients and 988 pulmonary nodules and compared the clinicopathological features and survival rates of patients with part-solid tumors to those of patients with pure GGOs and pure-solid tumors. The authors found that the prevalence of lymphatic involvement was lower in part-solid compared to pure-solid nodules, while the 5-year lung-cancer-specific RFS and OS of patients with part-solid tumors were worse than those of patients with pure GGOs but better than those of patients with pure-solid tumors [31]. Similarly, Uchida et al. found in their retrospective analysis that among patients (n = 177) who had undergone segmentectomy for clinical stage IA1-2-3 and IB NSCLC, as defined by the eighth edition of the TNM classification, pure-solid tumors were significantly associated with recurrence and worse survival, especially after 10 years, compared to non-pure-solid lesions [32]. Consequently, GGOs are the best candidates for segmentectomy. Patients with pure-solid nodules are expected to have higher recurrence, while in patients with part-solid nodules, tumor size and CTR may play a role in deciding the best surgical approach.

### 5.7. Maximum Standardized Uptake Value

A PET-CT scan is a routine radiological modality and is essential for helping the multidisciplinary team better assess and potentially predict tumor biology. The maximum standardized uptake value (SUV max) can be a potential indicator of aggressive tumor behavior, but the threshold above which it indicates more aggressive tumor behavior is a subject of scientific debate. In their retrospective study, Kocaman et al. analyzed 314 patients who had undergone wedge resection, segmentectomy, or lobectomy and found a relatively high SUVmax cutoff of 5.2 for pathological stage I NSCLC with a tumor size ≤  3 cm, above which lobectomy was found to be the better approach for RFS [33]. Huang L et al., in their aforementioned paper, defined an SUVmax cutoff of 4.5 or greater as an independent factor for nodal disease among the 478 included patients with clinical IA1-2 NSCLC patients (using the eighth edition of the TNM classification) [21]. Most commonly, an SUVmax cutoff of 2.5 is considered the threshold for hypermetabolic disease. Brunelli et al. analyzed the data of 164 patients after segmentectomy and 234 after lobectomy for peripheral clinical stage IA1-2 NSCLC; they defined hypermetabolic tumors as those with SUVmax greater than 2.5 and found better 4-year OS after lobectomy [34]. Similarly, Hurley and colleagues conducted a retrospective analysis of 527 patients with peripheral NSCLC ≤ 2 cm who had undergone either lobectomy or segmentectomy; they defined high-risk tumors as cT1b, solid-dominant, and hypermetabolic (SUVmax greater than 2.5) but found oncologically equivalent outcomes for high- and low-risk tumors [35]. On the other hand, Uchida and colleagues identified an SUVmax cutoff of 1.7 after analyzing the relationship between cases with and without recurrence in patients with clinical stage I NSCLC who had undergone segmentectomy. In their group with SUVmax ≥ 1.7, both 5- and 10-year OS significantly decreased and were associated with higher recurrence [30]. Thus, given the above-mentioned results, SUVmax can play a role in decision-making but might not be sufficient to decide operation strategy, and the cutoff value under which segmentectomy can be safely recommended without higher recurrence is still undergoing scientific research.

### 5.8. Surgical Margins

As for the technicalities of segmentectomy, the surgical margin itself and its adequacy remain among the most debated issues. Traditionally, thoracic surgeons have aimed to carry out a resection with a surgical margin of ≥2 cm or a margin-to-tumor ratio ≥ 1. Nakagawa et al., in their supplementary analysis of the JCOG0802/WJOG4607L trial data, identified that pure-solid appearance, a margin distance less than tumor size, and male sex were significantly associated with local recurrence, which occurred more frequently in patients with left lingular segment tumors compared to those with left upper division tumors [4]. However, in Kim et al.’s study of 156 patients with stage I lung cancer, after segmentectomy with a resection margin of less than 2 cm, recurrence developed in 10.7% of cases, with an RFS of 88.9%, and the parenchymal margin distance did not significantly affect the long-term prognosis. The isolated local recurrence rate was found to be only 1.9% [36]. Similarly, Donalgic et al. published a single-center retrospective study analyzing the data of 291 patients with cT1a-b-c cN0 NSCLC who had undergone segmentectomy; the authors found extremely low recurrence rates—1% for a median surgical margin of 13 mm and 32% for less than 10 mm. In their study, RFS was significantly associated with PET SUVmax greater than 3, pleural invasion, and nodal upstaging [37]. Given these somewhat contradictory results, surgical margin adequacy appears to be closely linked to tumor biology and not just a purely geometric parameter. Nevertheless, according to the European guidelines for the surgical management of pure ground-glass opacities and part-solid nodules, published by Cardillo and colleagues, in cases of sublobar resections and GGOs or part-solid nodules, a surgical margin ≥ 1 cm or a margin-to-tumor ratio ≥ 1 is recommended to ensure complete resection [38]. However, given the excellent general outcomes of patients with pure GGOs, a surgical margin even smaller than 1 cm might be sufficient. In Yoshino et al.’s study analyzing the long-term prognosis of patients with peripheral small GGOs after sublobar resection (258 wedge resection and 56 segmentectomy), the median surgical margin was 15 mm (from 0 to 60 mm), and local recurrence at the resection margin was found in only 1 (0.3%) of 314 enrolled patients 8.3 years after surgery. In their methodology, the macroscopic surgical margin was determined to be 5 mm or more when wedge resection was selected as the first choice. If a positive margin was detected by a frozen section after wedge resection, a conversion to segmentectomy was performed, and local recurrence was still only 0.3% on the surgical margin during their follow-up period [39]. According to these results, for pure GGO tumors, surgical margins of less than 1 cm but greater than 5 mm could prove to be sufficient to reduce the risk of local recurrence at the resection margin, but naturally, individual evaluation and a surgical decision regarding the applicability of wide wedge resection vs. segmentectomy should be considered on a case-by-case basis.

### 5.9. Intraoperative Frozen Section

Intraoperative frozen section of the suspected pulmonary nodule or hilar lymph node—when possible—greatly aids thoracic surgeons in borderline cases. In an article by Yeh et al., frozen sections and permanent slides from 361 resected stage I lung adenocarcinomas ≤ 3 cm were reviewed, and they found that frozen section had high specificity for micropapillary and solid patterns, but its sensitivity was low for these patterns: 37% and 69%, respectively. The ability to predict the dominant adenocarcinoma subtype pattern by frozen section is limited [40]. Moreover, in cases of simple segmentectomies, a wedge resection for frozen section might compromise the technical resection of the remnant segment afterwards. According to the ESTS consensus recommendation for technical standards by Brunelli and colleagues, frozen section from the segmental lymph nodes, however, should be performed in all cases except pure GGO lesions or compromised segmentectomies. In case of positivity, the operation should be expanded to lobectomy [16]. Despite this recommendation, intraoperative frozen section might not be available in all institutes, and its diagnostic accuracy for nodal involvement may not be undisputed. Huang L et al. found that patients whose preoperative tumor SUVmax was lower than 4.5 had only 2.2% nodal upstaging following segmentectomy and had comparable recurrence and survival to those of pN0 cases. Consequently, while frozen section accuracy might be 85%, it may only be necessary for highly metabolic tumors—with SUVmax ≥ 4.5 [21]. On the other hand, the role of frozen section in identifying STAS status might be considered as well. In Ding and colleagues’ retrospective analysis of 352 patients with clinical stage I NSCLC (≤2 cm), the accuracy of frozen section for STAS was 74%, while sensitivity was only 55% and specificity was 85% [41]. In the previously described study by Eguchi et al., the authors found the sensitivity of frozen section for STAS to range from 59% to 86% across five pathologists, with an observed percent agreement of 75.4%, and concluded that the application of frozen section may be a useful intraoperative tool in selecting the most appropriate resection for early-stage lung adenocarcinomas [27]. Still, despite the varying results for sensitivity and specificity, the use of frozen section for pulmonary nodules and suspicious hilar or mediastinal lymph nodes is an essential tool in the hands of thoracic surgeons, helping them characterize tumor biology during operation and better select patients for either segmentectomy or lobectomy.

### 5.10. Indocyanine Green (ICG) for Intersegmental Planes

During segmentectomy, discovering the correct intersegmental plane can be challenging, and the inflation test alone might be misleading owing to intersegmental inflation. However, the use of ICG can improve the accuracy of segmental resection. Yotsukura et al. retrospectively analyzed the data of 209 patients who had undergone segmentectomy using ICG. Good intersegmental demarcation was detected in 88% of cases, and no ICG-related adverse events were found. They also used high-frequency jet ventilation in 160 cases to visualize the intersegmental inflation–deflation plane and found that inflation spread beyond the intersegmental plane marked by ICG [42]. Consequently, the use of ICG—if available—can greatly facilitate adequate segmental resection, especially for surgeons with less experience. Although direct evidence demonstrating reduced locoregional recurrence with ICG-guided segmentectomy remains limited, several studies suggest that ICG may improve anatomical precision and margin accuracy during segmentectomy. Yotsukura et al. demonstrated superior intersegmental plane delineation using ICG fluorescence imaging [42], while Matsuura et al., in their phase II, prospective, single-center analysis of only 20 patients, reported excellent long-term oncologic outcomes with no local recurrence at the resection site following ICG-guided segmentectomy [43]. These findings, however limited, suggest that improved visualization of intersegmental planes may contribute to enhanced local control, although prospective comparative studies evaluating recurrence endpoints are still lacking.

### 5.11. Three-Dimensional Surgical Planning (3D)

Alongside ICG, the use of preoperative 3D planning and/or 3D printing is an undisputedly valuable tool in the hands of thoracic surgeons. In a retrospective analysis of 65 patients who had undergone either general segmentectomy or segmentectomy with the use of three-dimensional computed tomography bronchography and angiography (3D-CTBA) combined with a three-dimensional printing (3D printing) model, Hu et al. found that the operation time was significantly shorter and blood loss was lower in the 3D-CTBA group [44]. Three-dimensional modeling has an edge over hook-wire localization, in addition to not being an invasive procedure. Hong et al. compared the results of 3D reconstructions to hook-wire segmental resections in propensity-score-matched groups of 79 patients each and found that the hook-wire group had more complications, while the 3D reconstruction group had none, and the operative time, intraoperative bleeding, and postoperative chest tube duration were significantly reduced [45]. Three-dimensional reconstruction helps in identifying segmental structures and anatomical variation, better prepares the surgeon, and assists in the learning process for less experienced thoracic surgeons. To examine the possible impact of 3D planning on local recurrence, Lin and colleagues carried out a retrospective analysis of 281 enrolled patients with clinical stage IA2 NSCLC who had undergone surgical resection with or without the use of preoperative simulation. They found that patients with preoperative simulations had fewer relapses (2%) than patients without preoperative simulations (11.5%, *p* < 0.01); in the simulation group, only 1 patient had locoregional recurrence, while in the nonsimulation group, 15 patients developed locoregional recurrence during their follow-up period [46]. Although prospective randomized evidence remains limited, current data support the growing role of 3D planning in optimizing local control after segmentectomy.

### 5.12. Patient Characteristics and Functional Suitability

In cases of early-stage lung cancer, segmentectomy has a greater potential for lung preservation than lobectomy, but the extent is debatable. In the JCOG0802 trial, the lobectomy group experienced a median forced expiratory volume in 1 s (FEV1) reduction of 12%, while the segmentectomy group suffered an FEV1 loss of 8.5%, with the absolute difference being only 3.5%, which is marginal [2]. However, Wang X. et al.’s large meta-analysis of 13 studies comprising 2027 patients found that segmentectomy was associated with significantly better preservation of both FEV1 and %FEV1 compared to lobectomy [47], while Brascia et al.’s dual-center retrospective analysis showed a significant correlation between reduced DLCO and local recurrence [7]. In addition to intentional segmentectomy, thoracic surgeons are sometimes forced to perform compromised segmentectomy instead of lobectomy when, owing to patient characteristics, comorbidities, or insufficient lung functional reserve, the patient is not fit to undergo lobectomy, even for early-stage ≤ 2 cm pulmonary nodules. In this case, segmentectomy is considered when lobectomy would leave insufficient predicted postoperative reserve, especially when predicted postoperative FEV1 and/or predicted postoperative DLCO would fall below 30 or 40%, provided that the patient accepts the oncologic trade-offs and the tumor is technically suitable for the resection. In the consensus document of The American Association for Thoracic Surgery, Pennathur and colleagues aimed to define high-risk patients for lobectomy, but according to their consensus, no single preoperative FEV1 cutoff exists, and defining one might be misleading for patients with emphysema; they advise that postoperative predicted FEV1 and DLCO values be borne in mind when choosing the surgical method [48]. Other patient characteristics play a role in patient selection besides pulmonary function. Nakagawa et al.’s supplementary analysis revealed that male sex was significantly associated with local recurrence, while age was similar between the two groups [4]. Lopez et al. performed an observational study using 3533 patients in the registry of Spanish VATS Group and found no significant correlation between patient age, sex, and comorbidities and postoperative outcome or recurrence, whereas Brown and colleagues found a lower RFS and 5-year survival among patients with prior lung resections compared to those undergoing primary segmentectomies, though the differences were not statistically significant [9,18]. Suzuki et al. carried out a retrospective study of 1363 patients who had undergone lobectomy (n = 954) or segmentectomy (n = 409) for clinical T1N0M0 NSCLC. Patients with second primary lung cancer after previous lung malignancy were included. Patients who underwent segmentectomy for their first primary lung cancer had a higher probability of undergoing anatomical lung resection for their secondary primary lung cancer [49]. This underlines the importance of this approach in early-stage lung tumors. In the same study, smoking status played a significant role in developing second primary lung cancer: 17.9% were current smokers, 11.1% were past smokers, and 3.9% were non-smokers in this group. Additionally, squamous cell carcinoma as the first primary lung cancer and current smoking status were associated with an increased risk of developing secondary primary lung cancer. Patients in the segmentectomy group were older, but this difference remained on the verge of significance. Interestingly, among patients with secondary primary lung cancer, OS did not differ between surgical resection and stereotactic body radiotherapy/ablation [49].

### 5.13. Quality of Life (QoL) After Segmentectomy vs. Lobectomy

Beyond oncologic outcomes, preservation of postoperative QoL represents an important consideration when selecting the extent of pulmonary resection. Given that lung resection inevitably reduces functional pulmonary reserve, differences in respiratory symptoms, physical functioning, and overall health-related QoL may influence the relative benefits of segmentectomy and lobectomy. Several studies have suggested that segmentectomy may provide superior postoperative QoL compared with lobectomy, particularly regarding respiratory symptoms and physical resilience. Jiang et al. found that, among the 347 patients enrolled in their retrospective study, those who had undergone video-assisted sublobar resection (subsegment, segment, or wedge) showed a significantly better postoperative QoL in chest tightness, shortness of breath, cough, and expectoration compared to the lobectomy group [50]. Furthermore, perioperative complications and QoL were investigated in a phase III prospective randomized multicenter trial by Stamatis et al., where 108 patients with NSCLC (up to 2 cm) were included (54 segmentectomies and 54 lobectomies). The authors found that 12 months after surgery, patients who had undergone segmentectomy showed significantly less deterioration in physical and cognitive functioning and less dyspnea and fatigue. Moreover, recovery from dyspnea was found to be significantly faster in the segmentectomy group compared to lobectomy group [51]. In a retrospective study by Gómez-Martínez et al., 169 patients who had undergone segmentectomy (40.9%), lobectomy (56.2%), bilobectomy (0.9%), or pneumectomy (1.8%) were followed for 36 months. Interestingly, thoracotomy was performed in 69.2% of the cases, and only the remaining 30% were video-assisted operations. In this study, fatigue during walking was significantly less common after sublobar resections (55.1% vs. 72.7%). However, other than that, segmentectomy patients reported the same QoL regarding work, family, and social activities as those who had undergone more extensive lung resections [52]. Although several studies reported comparable long-term QoL outcomes between segmentectomy and lobectomy, the available evidence suggests that segmentectomy may facilitate faster recovery and improved preservation of physical functioning and respiratory well-being in appropriately selected patients with early-stage NSCLC.

After carefully reviewing available results from the literature, we aimed to identify a potential target patient group in which local recurrence after segmentectomy would be expected to not exceed that after lobectomy, using the primary guiding principle for segmentectomy provided by Brunelli and colleagues in the ESTS expert consensus recommendations [16]. However, weighing the findings of the contemporary literature collected above, we find that selecting patients and understanding and adequately interpreting patient- and tumor-specific preoperative or intraoperative findings remain challenging, and choosing the best surgical resection approach depends not only on a correct preoperative evaluation but also on the availability of technical aids such as preoperative 3D planning, ICG, frozen sections, robotic platforms and, of course, surgical expertise in the field of anatomical lung segmentectomies.

In Table 1, we have compiled the major predictors of local recurrence after segmentectomy for early-stage NSCLC, while in Table 2, we have attempted to collect and recommend potential diagnostic, surgical, or technical strategies to reduce local recurrence after segmentectomy.

## 6. Discussion

Several factors and limitations of different study designs make it difficult to synthesize the findings of two of the most cited prospective randomized trials (JCOG0802/WJOG4607L and CALGB/Alliance 140,503) and the results of several retrospective studies regarding the applicability and safety of anatomical lung segmentectomy in patients with nodal-negative clinical T1a-b- or even T1c-sized NSCLC.

Patient selection typically starts with an adequate and thorough patient-screening methodology and sensitive radiological evaluation of suspected GGOs—part-solid or small solid pulmonary nodules. A low-dosage CT screening program plays a pivotal role in early lung cancer management; roughly 80% of diagnosed cases have stage I or stage II disease, and of these, over 50% are stage IA [55]. Therefore, with the expansion of low-dosage CT screening programs, more than half of diagnosed patients have to be operated on by thoracic surgeons with the best knowledge to decide which patients are eligible for intentional segmentectomy as a sufficient surgical approach and in which cases lobectomy is the best option.

When selecting optimal candidates for segmentectomy, after patient screening, multiple preoperative evaluation and diagnostic procedures should be carried out to better assist the multidisciplinary team in deciding on the correct surgical strategy. A PET scan is a routine additional radiological diagnostic procedure that measures the uptake of FDG by the suspected malignant pulmonary nodule. Multiple studies have shown that preoperative SUVmax has prognostic value regarding patient survival and local recurrence. Interestingly, Uscida and colleagues found a low SUVmax ≥ 1.7 to be associated with a significantly higher recurrence rate and worse survival in their subgroup analysis [32]. In another study, Sun et al. analyzed the data of 200 patients who had received lobectomies for cT1N0M0 lung adenocarcinoma. They identified a preoperative SUV < 2.5 as a prognostic variable for lower lymph node metastasis and suggested that these patients could be treated with a segmentectomy [56]. The aforementioned study by Huang et al. determined a higher SUVmax ≥ 4.5 (versus <4.5) as the only independent factor for nodal involvement after segmentectomy [21]. Meanwhile, in Aboukheir and colleagues’ retrospective analysis, 102 patients who had undergone robotic-assisted segmentectomy and a preoperative PET-CT scan were divided into low SUV < 5 and high SUV ≥ 5 groups, and significant differences in OS, RFS, tumor histology, tumor grade, lymphovascular invasion, visceral pleural invasion, and recurrence were identified between the two groups, [57]. Therefore, SUVmax alone may be insufficient to determine the probability of recurrence and safely choose between segmentectomy and lobectomy as a surgical approach.

Another available tool to expand preoperative knowledge of small lung lesions is navigational bronchoscopy and guided biopsy from the nodule, which can be used to explore tumor biology, assess the risk of potential recurrence, and aid surgical planning. According to one study, the diagnostic rate of navigational bronchoscopy and subsequent biopsy of early-stage lung cancer was 79%, compared to 73.9% for transthoracic biopsy, with pulmonary nodules 10–30 mm in diameter [53].

Robotic-assisted navigation bronchoscopy is a recently developed method used in advanced thoracic centers to improve the accuracy and success rate of biopsies. In a large meta-analysis of 23 articles, comprising 1409 patients and 1540 nodules, Pyarali and colleagues found that for tumor sizes ranging from 5.9 to 25 mm, the pooled diagnostic success rate was 81.9% and the sensitivity for malignancy was 87.6%, with minimal incidence of complications [54]. With the aid of this advanced, minimally invasive robotic-assisted system, the localization and success rate of biopsies from small pulmonary nodules are substantially better, as is patient safety, providing important pathohistological data on the tumor that can help in decision-making regarding the surgical approach.

Although preoperative screening and diagnostic tools are not inherently linked to thoracic surgery, they are important topics of discussion in this field: centers increasingly have access to these new technologies and, after successfully screening and diagnosing a small pulmonary lesion, even if smaller than 8 or 10 mm in diameter, an understanding of the tumor biology—beyond its sheer size and radiomorphology—can begin to guide the surgical team in adequately choosing between segmentectomy and lobectomy. Accordingly, multiple studies found that higher-grade early-stage NSCLC tumors with solid or micropapillary dominance have a higher tendency toward local recurrence or nodal involvement after segmentectomy [13].

The presence or absence of STAS is also a well-established guiding factor, with several studies finding that STAS-positive tumors have higher recurrence rates after segmentectomy compared to lobectomy [26].

Tumor size per se and radiological appearance alone can also be guiding factors. A retrospective analysis of 988 pulmonary nodules found that 5-year lung-cancer-specific RFS and OS of patients with part-solid nodules were worse than those of patients with pure GGOs and better than those of patients with pure-solid tumors. CTR and tumor size could predict invasiveness for patients with part-solid nodules but could not predict the prognosis [31]. In the previously mentioned retrospective study by Hattori et al., pure-solid radiological appearance and tumor size were significant predictors of locoregional recurrence. Recurrence was 20.7% after segmentectomy and 8.2% after lobectomy. Furthermore, segmentectomy and larger tumor size were independent significant factors for recurrence in their multivariate analysis [11]. Still, the future of segmentectomy is likely determined not by tumor size alone but by accurate integration of radiologic, biological, metabolic, and molecular risk factors.

Different surveillance strategies should be used for lesions smaller than 1 cm after segmentectomy, given that in the previously mentioned article by Wada and colleagues, late recurrences could occur beyond 4 years after surgery, even in tumors with a diameter less than 1 cm. Five-year follow-up strategies and OS and RFS data might render segmentectomy comparable to lobectomy, but more studies comparing 10-year OS and RFS data of selected patient groups are required to reveal and further understand the limiting factors of segmentectomies. Surveillance beyond 5 years is mandatory; long-term CT follow-up is strongly recommended.

## 7. Conclusions

Segmentectomy has fundamentally changed the surgical management of early-stage NSCLC and has evolved from a compromised procedure for high-risk patients into an intentional oncologic operation for carefully selected tumors. The available evidence demonstrates that segmentectomy can provide survival outcomes comparable to those achieved with lobectomy in selected patients; however, local recurrence remains its principal oncologic limitation. The contemporary literature consistently highlights the strong influence of tumor biology on recurrence risk, including pure-solid radiological appearance, a high consolidation-to-tumor ratio, aggressive adenocarcinoma subtypes, STAS positivity, lymphovascular invasion, and occult nodal disease. Male sex has also been identified as a predisposing patient characteristic linked to increased local recurrence. Technical factors such as inadequate lymph node assessment, insufficient surgical margins, and anatomical complexity of resections further contribute to recurrence risk. Importantly, several studies have demonstrated delayed locoregional relapse occurring beyond 5 years after surgery, emphasizing the necessity of prolonged postoperative surveillance. The recent long-term reassessment of randomized trials, particularly JCOG0802/WJOG4607L, suggests that enthusiasm surrounding universal application of segmentectomy should be moderated, especially for biologically aggressive tumors. Consequently, segmentectomy should be considered an oncologically demanding procedure that requires meticulous surgical technique, comprehensive nodal evaluation, and careful, biology-driven patient selection.

## 8. Future Directions

Future progress in the field of segmentectomy will likely focus on individualized, biology-driven surgical decision-making rather than relying solely on tumor size and radiomorphological criteria. Advances in radiomics, artificial intelligence, and machine learning-based imaging analysis may improve the preoperative prediction of aggressive tumor behavior, including STAS positivity, occult nodal disease, and high-grade histology. Molecular profiling and genomic characterization of early-stage NSCLC may further refine patient selection by identifying tumors with increased recurrence potential despite their small size. The increasing use of navigational bronchoscopy and robotic-assisted bronchoscopic biopsy platforms may provide more accurate preoperative histopathological diagnosis and improve surgical planning. Additionally, modern intraoperative technologies such as indocyanine green fluorescence imaging, three-dimensional reconstruction, augmented reality-assisted surgical navigation, and the careful use of frozen sections may enhance anatomical precision, margin assessment, and surgical planning during operation. Future prospective studies with long-term follow-ups are required to better define the optimal indications for segmentectomy, particularly in patients with pure-solid tumors, high-SUVmax lesions, and aggressive adenocarcinoma subtypes. Ultimately, the integration of tumor biology, imaging characteristics, molecular markers, and advanced surgical technologies may enable a truly personalized surgical strategy, whether segmentectomy or lobectomy, for early-stage NSCLC.

## Figures and Tables

**Table 1:** Major Predictors of Local Recurrence after Anatomical Segmentectomy for Early-Stage NSCLC.

Variable	Reference	Local Recurrence/Oncologic Outcome After Segmentectomy	Clinical Implication
STAS-positive	Kadota et al. [26]	5-year CIR: 42.6%; locoregional recurrence: 22.2%	Lobectomy preferred
STAS-negative	Kadota et al. [26]	5-year CIR: 10.9%; locoregional recurrence: 4.1%	Favorable candidates for segmentectomy
SUVmax ≥ 2.5	Brunelli et al. [34]	Hypermetabolic tumors associated with better oncologic outcomes after lobectomy	Careful patient selection and nodal assessment recommended
SUVmax ≥ 4.5	Huang et al. [21]	Higher nodal involvement (OR 3.5); increased recurrence risk	Systematic lymph node dissection should be considered
SUVmax ≥ 5.2	Kocaman et al. [33]	Worse RFS after segmentectomy	Lobectomy may provide superior oncologic control
SUVmax < 1.7	Uchida et al. [32]	Significantly improved 5- and 10-year OS and lower recurrence	Excellent candidates for segmentectomy
Margin distance < tumor size	Nakagawa et al. [4]	Significantly associated with local recurrence	Adequate margins are critical during segmentectomy
Margin-to-tumor ratio < 1	Eguchi et al. [27]	Significantly increased locoregional recurrence (7%)	Extended margins or lobectomy should be considered
Margin ≥ 2 cm	Kim et al. [36]	Low isolated local recurrence (1.9%)	Wide parenchymal margins improve local control
CTR > 0.5 (solid-dominant)	Wada et al. [5]	Higher locoregional recurrence after segmentectomy (13.9% vs. 7.9%)	Solid-dominant tumors require cautious selection
CTR ≥ 0.86	Kamigaichi et al. [12]	Independent predictor of recurrence	High CTR indicates aggressive tumor biology
CTR < 0.5	Nakagawa et al. [4]	Lower local recurrence risk	Favorable indication for segmentectomy
Pure GGO	Woo et al. [30]	Associated with favorable histology and low recurrence	Excellent candidates for segmentectomy
Part-solid nodules	Ye et al. [31]	Intermediate recurrence risk between GGO and pure-solid lesions	Individualized decision- making recommended
Pure-solid nodules	Hattori et al. [11]	Locoregional recurrence: 20.7% after segmentectomy vs. 8.2% after lobectomy	Lobectomy may be preferred in biologically aggressive tumors
Tumor size ≥ 1.5 cm	Vakouftsi et al. [10]	5.1-fold higher recurrence risk	Larger than 1.5 cm tumors require careful oncologic evaluation
Lymphovascular invasion	Brascia et al. [7]	Predictor of regional recurrence	Indicates biologically aggressive disease
Micropapillary/solid subtype	Xu et al. [13]	Significantly associated with recurrence	Lobectomy preferred
≥6 harvested lymph nodes	Huang et al. [19].	Improved 3-year RFS: 90.2% vs. 73.7%	Adequate nodal assessment improves oncologic outcomes
<6 harvested lymph nodes	Huang et al. [19].	Increased recurrence risk	Insufficient nodal staging may contribute to occult disease
Lack of 3D planning	Lin et al. [46]	Higher recurrence without preoperative 3D simulation (11.5% vs. 2.0%)	Preoperative 3D planning may improve oncologic precision
ICG-guided segmentectomy	Matsuura et al. [43]	No local recurrence at resection margin reported	Improved intersegmental visualization may enhance local control

**Table 2:** Potential Strategies to Reduce Local Recurrence after Anatomical Segmentectomy for Early-Stage NSCLC.

Strategy	Supporting Evidence	Potential Benefit
Systematic hilar and mediastinal lymph node dissection	Huang et al. [19]; Tudor et al. [15]; Hattori et al. [11]	Improves occult nodal disease detection, reduces understaging, and may decrease locoregional recurrence
Intraoperative frozen section of hilar/intersegmental lymph nodes	Yeh et al. [40]; Brunelli et al. [16]	Enables intraoperative identification of occult nodal disease and conversion to lobectomy when necessary
Intraoperative STAS assessment by frozen section	Eguchi et al. [27]	May identify high-risk tumors unsuitable for segmentectomy
Adequate surgical margins (margin ≥ 2 cm or margin-to-tumor ratio ≥ 1)	Nakagawa et al. [4]; Kim et al. [36]; Eguchi et al. [27]	Reduces isolated local recurrence and margin failure
PET-based patient selection (SUVmax-guided)	Uchida et al. [32]; Kocaman et al. [33]; Huang et al. [21]	Identifies biologically aggressive tumors and occult nodal disease preoperatively
Careful selection of GGO- dominant/low CTR tumors	Hattori et al. [11]; Kamigaichi et al. [12]; Nakagawa et al. [4]	Improves oncologic suitability for segmentectomy
STAS-oriented surgical decision-making	Kadota et al. [26]; Eguchi et al. [27]	Lobectomy may prevent recurrence in STAS-positive tumors
Three-dimensional (3D) reconstruction and preoperative planning	Lin et al. [46].; Xu et al. [13]	Improves anatomical precision, margin assessment, and may reduce recurrence
Indocyanine green (ICG)-guided segmentectomy	Yotsukura et al. [42]; Matsuura et al. [43]	Improves intersegmental plane visualization and margin accuracy
Navigational bronchoscopy and robotic-assisted biopsy	Lentz et al. [53]; Pyarali et al. [54]	Improves preoperative diagnostic accuracy and biological characterization
Complex segmentectomy performed in experienced centers	Handa et al. [29]	Reduces technical errors and inadequate resections
Systematic postoperative surveillance beyond 5 years	Nakagawa et al. [4]; Hattori et al. [11]	Detects delayed locoregional recurrence, particularly in solid-dominant tumors
Functional risk stratification (ppoFEV1, DLCO, CPET)	Brunelli et al. [16]; Pennathur A et al. [48]	Identifies patients who may benefit from compromised segmentectomy while balancing oncologic risk

## Data Availability

No new data were created or analyzed in this study.

## Abbreviations

The following abbreviations are used in this manuscript:

CT	Computer Tomography
CTR	Consolidation-to-tumor ratio
CIR	Cumulative incidence of recurrence
DFS	Disease-free survival
DLCO	Diffusing capacity of carbon monoxide
ESTS	European Society of Thoracic Surgeons
FEV1	Forced expiratory volume in 1 s
GGO	Ground-glass opacity
IASLC	International Association for the Study of Lung Cancer
ICG	Indocyanine green
SUVmax	Maximum standardized uptake value
NSCLC	Non-small cell lung cancer
OS	Overall survival
PET	Positron emission tomography
QoL	Quality of life
RFS	Recurrence-free survival
STAS	Spread through air spaces
